# Œsophageal Stricture

**Published:** 1888-10

**Authors:** E. T. Painter

**Affiliations:** Pittsburg, Penn.


### ŒSOPHAGEAL STRICTURE.*

E. T. PAINTER, M. D., PITTSBURG, PENN.

The patient, aged about thirty-eight, complained of difficulty in swallowing food and its regurgitation, both liquid and solid, and of inability to drink cold water or other cool drink. It was first noticed that food would not move on in response to the usual movement of deglutition, and that its onward progress was assisted by a few gentle raps on the back. This symptom first

*A paper read at Allegheny County Medical Society.

showed itself about six years ago. From this slight difficulty in the passage of solid food to the stomach, the patient gradually found herself compelled to subsist wholly on liquid foods, and these could be retained only when taken at a certain warm temperature. Neither water at ordinary temperature, nor cool drink of any sort, nor solid food, had entered the stomach in a period of years. She was emaciated and destitute of physical vigor.

An examination of the œsophagus with a bougie proved the existence of a band, which could resist the further progress of the instrument till the constriction *willed* to give way, when the bougie could easily slip into the stomach. Neither had the diameter of the bougie, nor the flexibility of a tube, nor force, seemed to have anything to do with passing through the constricting ring. Passage beyond the constriction could be made only when the ring was so disposed and inclined. There had been no pain or hæmorrhage. There was no history of the introduction of a foreign body and its impaction, or of the swallowing of a strong acid or strong alkali. No aneurism was evident. There is no history of carcinoma. The constriction was sixteen inches from the lower incisors. Dysphagia and regurgitation, which prevented the patient retaining sufficient nourishing food, were the only symptoms given.

As drugs, massage, the passage of a flexible tube and the Faradic current had failed to accomplish any good results, I resolved to give a thorough trial to the galvanic current locally applied, and that experiment I proceeded to make. I employed from six to ten cells of a galvanic chloride of silver battery, placing a sponge electrode joined to the negative pole in one hand, and an œsophageal electrode connected with the positive pole within the constricting ring. This electrode consisted of an ovoid shell, seven-sixteenths of an inch by three-fourths of an inch of perforated hard rubber, which could be unscrewed in the middle, and had sufficient space within for absorbent cotton which came in contact with a small expanse of platinum, and that in turn was united by an insulated wire to the battery.

The battery used gives a current absolutely constant in character, and a water rheostat served to differentiate the strength of the current. My applications were made three times a week for a few weeks; then twice a week, each treatment lasting from six to twelve minutes.

At the termination of the first treatment the following contingent took place: The current had passed for as long as I thought best, when, on attempting to withdraw the œsophageal electrode, it came easily in response to my traction for a few inches, when it was seized by a contraction of the œsophagus and there firmly held for a few seconds. This peculiar accident did not happen a second time. After each treatment the patient placed herself in a recumbent position for a half hour. At the fourteenth visit the electrode was passed through and beyond the point of stricture without the knowledge of the patient, nor did I feel any sensation of opposition. At dinner, after the fifteenth application, the patient ate meat and bread and butter.

In about three months I made twenty-five applications, obtaining most decided improvement. The contracting ring persists, but its irritability has disappeared. The patient eats, without regurgitation, of what others at the table partake, restricting herself in only one item of food, meat, which is cut fine for her. She drinks water and milk freely. For a time the stomach, unaccustomed to such foreign substances as bread and butter, strawberries, cheese, etc., made the patient aware of its change in function by dyspeptic disturbances.

The interesting points in this case are these: The ease with which a diagnosis of dyspepsia could have been made, its long existence, its obstinacy under manifold treatment, the continued presence of the stricture without its irritability, and the rapid change in character of the constriction under the influence of the galvanic current.

